# The crystal structure of monoacylglycerol lipase from *M. tuberculosis* reveals the basis for specific inhibition

**DOI:** 10.1038/s41598-018-27051-7

**Published:** 2018-06-12

**Authors:** Philipp Aschauer, Robert Zimmermann, Rolf Breinbauer, Tea Pavkov-Keller, Monika Oberer

**Affiliations:** 1 Institute of Molecular Biosciences, University of Graz, Humboldtstraße 50/3, 8010 Graz, Austria; 2 BioTechMed-Graz, Mozartgasse 12/II, 8010 Graz, Austria; 3 Institute of Organic Chemistry, Graz University of Technology, Stremayrgasse 9, 8010 Graz, Austria

## Abstract

Monoacylglycerol lipases (MGLs) are enzymes that hydrolyze monoacylglycerol into a free fatty acid and glycerol. Fatty acids can be used for triacylglycerol synthesis, as energy source, as building blocks for energy storage, and as precursor for membrane phospholipids. In *Mycobacterium tuberculosis*, fatty acids also serve as precursor for polyketide lipids like mycolic acids, major components of the cellular envelope associated to resistance for drug. We present the crystal structure of the MGL Rv0183 from *Mycobacterium tuberculosis* (mtbMGL) in open conformation. The structure reveals remarkable similarities with MGL from humans (hMGL) in both, the cap region and the α/β core. Nevertheless, mtbMGL could not be inhibited with JZL-184, a known inhibitor of hMGL. Docking studies provide an explanation why the activity of mtbMGL was not affected by the inhibitor. Our findings suggest that specific inhibition of mtbMGL from *Mycobacterium tuberculosis*, one of the oldest recognized pathogens, is possible without influencing hMGL.

## Introduction

Despite intense research over the last decades, tuberculosis caused by the pathogen *Mycobacterium tuberculosis* (Mtb) still remains one of the most deadly human diseases worldwide. The World Health Organization (WHO) estimates more than 9 million cases and approximately 1.8 million deaths in 2015, with approximately one-third of the population being infected with the bacterium^1^. Unfortunately, the increasing prevalence of tuberculosis strains resistant to one or more antibiotics (multi drug resistant tuberculosis) render current available treatment strategies ineffective. The WHO released a statement in February 2017 reaffirming the critical need for research on new antibiotics for drug resistant Mtb^2^.

Apart from drug-resistant strains, therapy of tuberculosis faces another enormous challenge, which is inherent to the mode of infection by Mtb. Initial infection with Mtb leads to the formation of characteristic granulomas where the pathogens remain viable and can evade the host immune system for decades. This dormancy period is characterized by downregulated metabolism associated with low susceptibility to anti-tuberculosis agents, which might cause the requirement for very long-term treatments. About 10% of the latently infected individuals will develop tuberculosis during their lifetimes. Therefore, it is of utmost importance for effective tuberculosis treatment to identify potential drug targets that remain accessible during the dormant phase of Mtb infection and are therefore susceptible for pharmacological intervention. When taken up by macrophages through phagocytosis, Mtb ends up in phagosomes where it cannot be processed due to its cell envelope. In the resulting Mtb infected macrophages, this pathogen-loaded phagosomes have been shown to directly interact with lipid-loaded droplets of the host cell^3,4^. If lipids are extracted from membranes or lipid droplets of the host cell during infection is still under debate^4–6^.

Genome sequencing of *M. tuberculosis* revealed an outstandingly high number of genes associated with lipid metabolism, indicative of a strong role of lipolytic genes in the various mycobacterial life stages^7^. Re-activation and transition to the infectious stage requires energy, which is likely derived from accumulated intracellular lipid inside the granuloma^8,9^. Consequently, enzymes involved in lipid metabolism of pathogenic bacteria have caught special attention of researchers as route towards the discovery of novel inhibitory therapeutics or biomarkers^10–13^.

Fatty acids (FAs), possibly derived from host cell lipids, have been known as an important source of nutrition for Mtb during infection for several decades^5^. FAs are used as energy source since they are substrates for β-oxidation. They are also incorporated into triacylglycerols which can be stored in intracellular lipid inclusions^5,8,14^ or into phospholipids which are crucial to maintain the integrity of the cytoplasmic membrane^5^. In Mtb, FAs also serve as precursors for polyketide lipids such as phiocerol-dimycoseroic acid, poly-acylated trehaloses, and mycolic acids. The first two are used as sinks to bind propionyl-CoA which would otherwise be toxic, the latter is used for the synthesis of the Mtb envelope^5,15,16^. Mtb is capable of taking up exogenously added FAs during a dormancy resembling state^8^. One possibility for the pathogen to recruit crucial FAs for membrane synthesis is via degradation of host cell lipids from lipid droplet stores and membrane phospholipids followed by assimilation of the resulting FAs.

Monoacylglycerol lipases (MGLs) resemble a major enzyme class involved in the hydrolysis of monoacylglycerols (MGs) to glycerol and FAs from intracellular and extracellular sources.

The protein encoded by the gene *Rv0183* from *Mycobacterium tuberculosis* (strain H37Rv) has been annotated as a MGL^6^ and is referred to as mtbMGL throughout this manuscript. mtbMGL is exported into the media in cultured cells and is therefore suggested to be involved in the supply of FAs to the pathogen by digestion of host cell lipids^6^. Consequently, inhibition of MGL in Mtb might reduce the ability of Mtb to take up host cell lipids. To our knowledge, no knock-out of the *Rv0183* gene is available. However, deletion of the homolog *Mycobacterium smegmatis* gene showed morphological changes, rendering mtbMGL an interesting target for weakening the pathogen and increasing its susceptibility for drug responsiveness^17^. Even more important, mtbMGL was shown to be expressed during latent and re-activated stages of Mtb infection^18^ thus increasing the chance that mtbMGL is indeed druggable even during the dormant phase of infection with the pathogen. Additionally, MGs *per se* have been shown to inhibit bacterial cell growth. Therefore inhibiting MG degrading lipases might have toxic effects onto bacteria^19,20^.

Three-dimensional (3D) structural information contributes immense know-how to rational drug design. In recent years, high resolution crystal structures of MGL orthologs from different organism including human, yeast and *Bacillu*s sp. H257 have become available^21–27^. Many research efforts in academia and industry aim for identifying a specific inhibitor for human MGL (hMGL) due to its effects on the endocannabinoid system, inflammation, cancer progression and pain sensation^28,29^. Obviously, structural comparison of hMGL and mtbMGL is of great value in the quest for specific mtbMGL inhibitors to avoid off-target effects, cross-reactivity, and for re-purposing of existing lead compounds.

The herein presented 3D structure of mtbMGL at 1.8 Å resolution provides the structural basis for rational design of drugs specifically targeting mtbMGL which opens a novel route in the demanding battle against tuberculosis.

## Results and Discussion

### The 3D structure of mtbMGL reveals an α/β hydrolase fold with an open cap conformation

**Figure 1:**
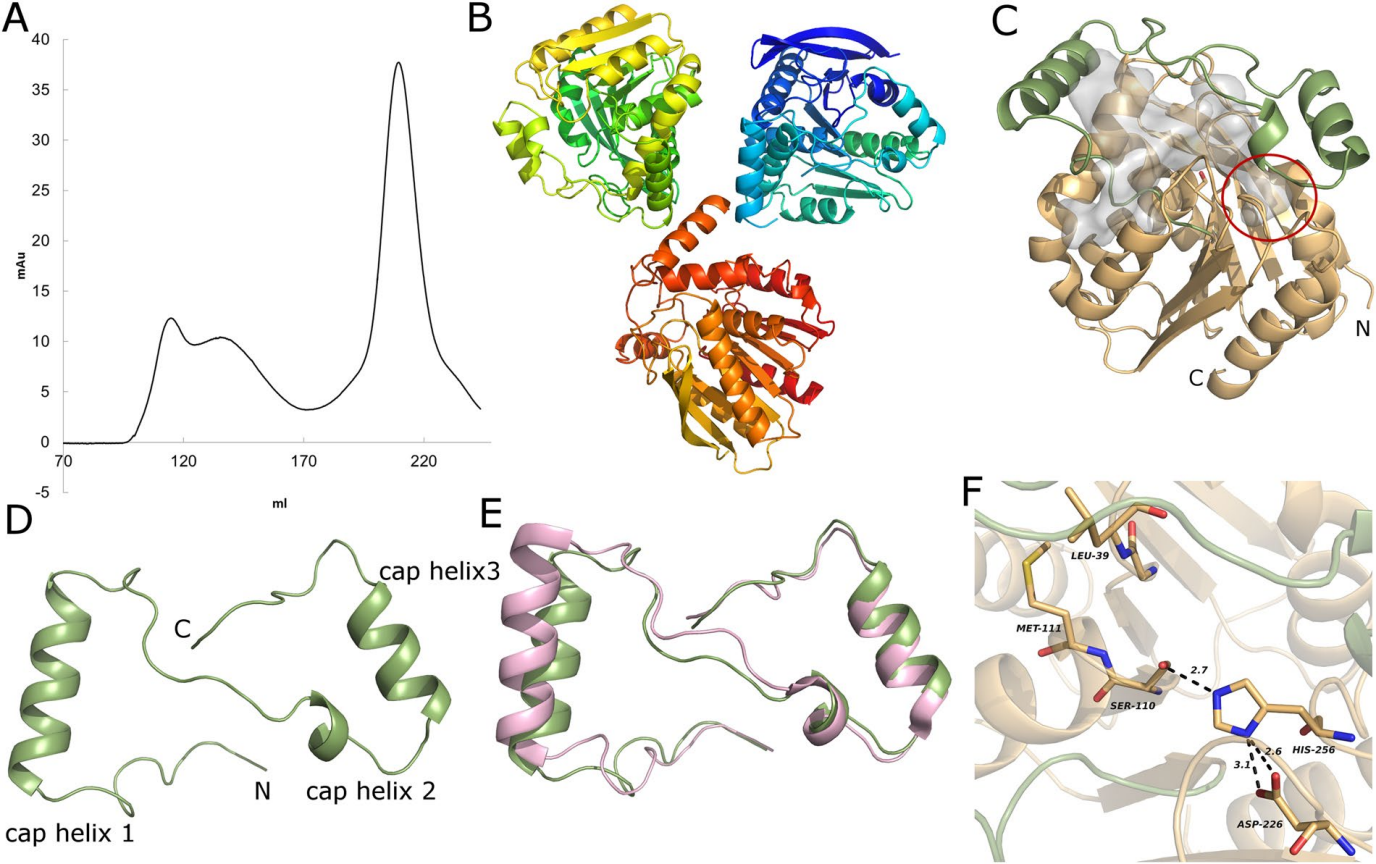
(**A**) Size exclusion chromatogram of mtbMGL after purification via Ni-NTA Agarose resin. The monomeric fraction (eluting at approximately 205 ml) was used for crystallization. (**B**) Asymmetric unit in the mtbMGL crystal. (**C**) Overall structure of mtbMGL (chain B), the cap region is in green and the α/β hydrolase core in wheat. The gray blob represents the cavity inside the protein calculated by CASOX. The small cavity accommodating the glycerol moiety is marked with a red circle. (**D**) Cap region of mtbMGL viewed from the top (compared to orientation in 1C). (**E**) Cap regions from hMGL and mtbMGL aligned to each other (hMGL in pink, mtbMGL in green. (**F**) Close up view on the active site of mtbMGL with the residues of the catalytic triad and the residues that build up the oxyanion hole shown as sticks.

Rv0183 was overexpressed in *E. coli* and isolated via affinity chromatography yielding highly pure protein (yield of approx. 8 mg per liter of culture). The monomer fraction was separated by SEC chromatography (Fig. 1A), concentrated and crystallized.

The crystals of mtbMGL diffracted to a resolution of 1.8 Å. The structure was solved by molecular replacement and refined to final R-values of R_work_ = 22.13% and R_free_ = 25.10%. The R values are high for of a high resolution data set yet can be explained by twinning (twin operator: h,k,-l, twin fraction 0.13) of the data set used for structure determination. Data collection and refinement statistics are summarized in Table 1. Three molecules of mtbMGL are present in the asymmetric unit with a helix formed by the residues Pro145-Val157 packed against the corresponding helix in the next protein chain (Fig. 1B).

**Table 1:** Measurement, processing and refinement statistics for the mtbMGL crystal structure.

Measurement and Processing						
Wavelength (Å)	0.97895					
Resolution (Å)	49.21 – 1.80 (1.84–1.80)					
Space group	*P* 3_1_ 1 2					
	a	b	c	α	β	γ
Unit cell (Å, °)	85.82	85.82	196.98	90.0	90.0	120.0
Total reflections	741011 (39737)					
Unique reflections	77304 (4549)					
R_merge_	0.105 (0.853)					
R_pim_	0.035 (0.307)					
R_meas_	0.111 (0.907)					
CC 1/2	0.998 (0.734)					
Completeness	100.0% (100.0%)					
Mean I/sd(I)	16.4 (2.6)					
Multiplicity	9.6 (8.7)					
Average Mosaicity	0.18°					
**Refinement**						
R_free_	0.251					
R_work_	0.221					
r.m.s.d. bonds (Å)	0.003					
r.m.s.d. angles (°)	1.010					
Ramachandran favored (%)	96.69					
Ramachandran disallowed (%)	0.00					

Numbers in brackets are for the high resolution shell.

mtbMGL harbors an α/β hydrolase core consisting of 8 β-strands surrounded by 6 α-helices (Met1-Val137 and Ala194-Leu279) and a cap region (Ala138-Pro193) containing 3 α-helices (Fig. 1C,D). The cap-region has a Z like shape which resembles the specific architecture observed in other structurally characterized MGLs (Fig. 1E)^21,22,25,30^. Residues Ser110, His256 and Asp226 form the active site in the core domain of mtbMGL (Fig. 1F). The nucleophile Ser110 within the consensus GXSXG lipase motif is located between strand 4 and helix 2 of the α/β hydrolase core. The backbone nitrogen atoms of Met111 and Leu39 form the oxyanion hole that can stabilize the transition state intermediate during the catalytic reaction (Fig. 1F). The catalytic center is located in an approx. 18 Å deep cavity and can be reached from the outside in the observed open conformation via entry between the first and second stretch of the cap region (residues Ala138 to Arg173, Fig. 1C). The nature of the substrate-binding cavity is governed by predominantly hydrophobic side-chains located in the cap and core domains: Leu39, Ala136, Val137, Ala138, Ala139, Leu142, Val143, Val147, Ala148, Ala15, Leu154, Leu160, Val163, Phe168, Ile171, Val192, Ala194, Leu200, Leu201, Leu228 and Ile229, Pro230. A smaller extension of the cavity reaches past the nucleophilic serine towards the N terminus of the protein (Fig. 1C, red circle).

### Docking studies reveal numerous interactions of mtbMGL to recognize polar and apolar segments of the MG target substrate

**Figure 2:**
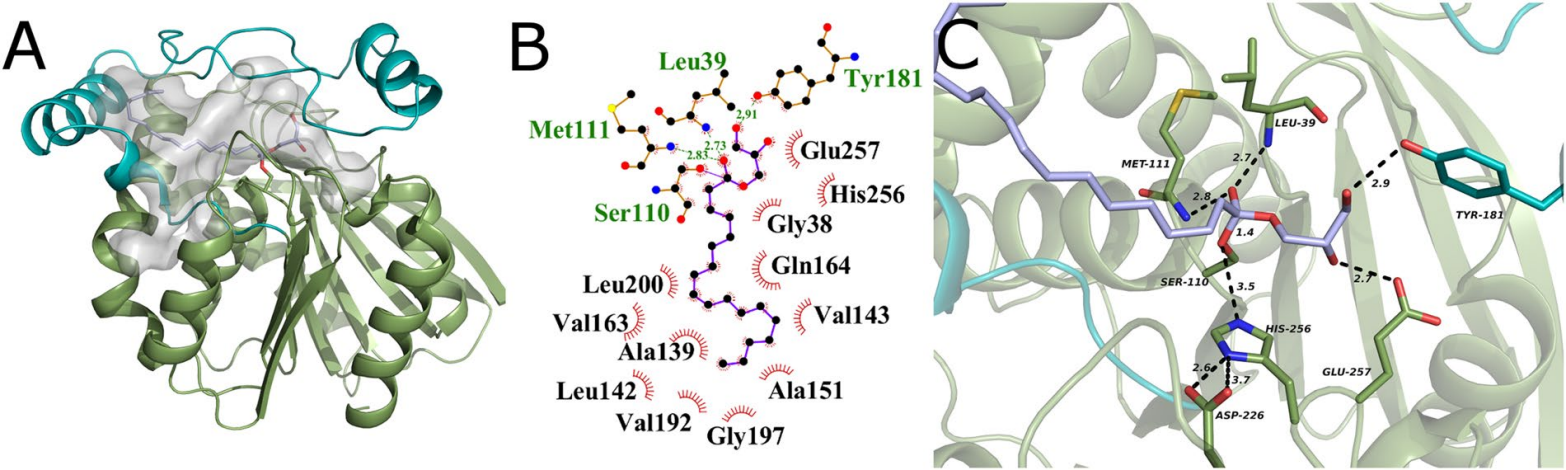
(**A**) Cartoon representation of the mtbMGL crystal structure with 1-OG docked. The cap is shown in cyan and the α/β hydrolase core in green. The gray blob shows the cavity inside the protein calculated omitting the ligand. Gray sticks show 1-OG covalently bound to the active site serine. (**B**) Ligplot+^48^ cartoon of all the residues interacting with the ligand. The hydrophobic interactions are shown as brown circle sections. (**C**) Close up view of catalytic triad and residues interacting with the glycerol moiety in the 1-OG docked mtbMGL crystal structure. Residues interacting with the glycerol moiety (Tyr181, Glu257) are shown as sticks.

Next, we were interested to investigate potential substrate binding in mtbMGL. Despite attempts towards co-crystallizing mtbMGL with substrate analogs, no crystals of a complex could be obtained at this point. Therefore, we performed docking studies with the substrate 1-oleoyl-glycerol (1-OG) in the form of the tetrahedral reaction intermediate (Fig. 2). We used only 1-OG for docking since no positional preference for sn-1(3) or 2-MGs has been reported for any MGL. In the best-docking mode, the polar glycerol moiety of the substrate is in direct interaction with catalytically important residues Ser110, His256, Glu257, Met111 and Leu39 from the core region and Tyr181 situated within helix 3 of the cap (Fig. 2B,C). The peptide NH groups of Met111 and Leu39 are in hydrogen bonding distance to the negatively charged oxygen atom of the tetrahedral intermediate (Fig. 2B,C). Hydrophobic interactions of the alkyl chain with residues Ala139, Leu142, Val143, Ala151, Val163, Val192, Gly197 and Leu200, keep the long carbon chain of the substrate in place and shielded from the aqueous environment (Fig. 2B). The glycerol head group of 1-OG is accommodated at the entrance to a small extension of the substrate binding cavity and forms hydrogen bonds to residues Tyr181 and Glu257 of the cap and the core, respectively.

### Differences in the binding pocket of mtbMGL compared to human MGL impair JZL-184 inhibition

**Figure 3:**
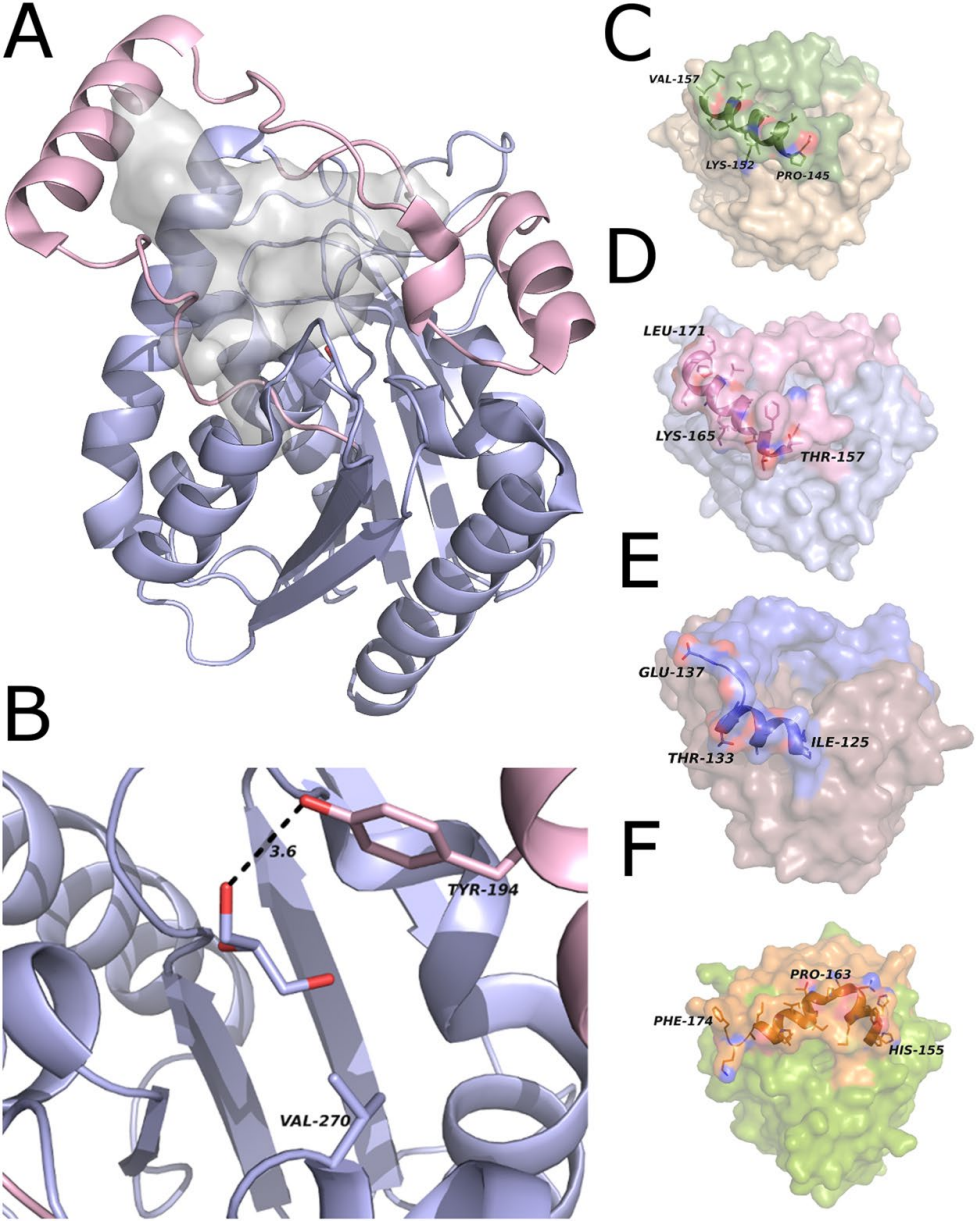
(**A**) Overall structure of hMGL (PDB ID 3HJU, chain A). The gray blob shows the cavity inside the protein. The cap is shown in pink and the core in light blue. (**B**) Close up view of the small cavity in hMGL (PDB ID 3HJU, chain A). The residues at equivalent positions to the glycerol moiety binding residues in mtbMGL are shown as sticks. (**C**) Surface of mtbMGL with the α/β-hydrolase core in wheat and the cap region in green. The side chains in cap helix 1 shown as sticks and the backbone in cartoon representation. (**D**) The surface of hMGL with the α/β-hydrolase core in blue and the cap region in pink. The side chains in cap helix 1 are shown as sticks and the backbone as cartoon (PDB ID 3HJU, chain A). (**E**) Surface representation of bMGL with the α/β-hydrolase core in brown and the cap in blue. The side chains in cap helix 1 are shown as sticks and the backbone as cartoon (PDB ID, 4KEA, chain A).(**F**) Surface view of the Yju3p structure with the α/β-hydrolase core in green and the cap region in orange. The backbone of cap helix 1 and cap helix 2 is shown as cartoon and the side chains as sticks (PDB ID 4ZWN, chain A).

MGLs are ubiquitous lipases found in prokaryotic and eukaryotic organisms. The structurally characterized MGLs share low sequence conservation, namely 34%, 21% and 23% sequence identity of mtbMGL, bMGL, and Yju3p to hMGL, respectively. Nevertheless, the sequential arrangement of secondary structure elements and the fold topology harboring a catalytic triad are highly conserved^21–25,31^ (Fig. S1). Along these lines, the Cα-trace root-mean-square-deviation of hMGL vs mtbMGL is only 1.03 Å. The close structural similarity of those two proteins is remarkable considering the low sequence identity and evolutionary distance between bacteria and humans (Figs 1C and 3A). Interestingly, even the overall Z-shape of the cap regions, which can differ vastly in length and secondary structure elements across MGL orthologs is highly conserved (Fig. 1E).

The hitherto reported MGL structures indicate great overall conservation of the cap architecture^30^. Conformational flexibility within this overall shape leads to closed and different open conformations, the latter even within different molecules of one asymmetric unit^21–26^. The here presented crystal structure of mtbMGL corresponds to an open conformation in all three molecules of the asymmetric unit and enables a direct access path of the substrate to the catalytic sites (Figs 1 and 2). The open conformations of mtbMGL and hMGL are very similar and differ from that of bMGL with respect to the elements mediating the conformational changes. The first helix in the cap region, referred to as cap helix 1 in this manuscript (Fig. 1D, Fig. S1), is the major player in the conformational changes of hMGL. This cap helix has a very similar position and length in mtbMGL (Pro145-Val157) and hMGL (Thr157-Leu171). This is remarkable since this part varies in secondary structure length and composition in other MGLs^21,22,24,25^ (Fig. 1E). In bMGL, the corresponding residues adopt a short cap helix 1 (Ile125-Thr133) followed by a loop region (Fig. 3E). In Yju3p, the MGL ortholog of S.*cerevisiae*, Pro163 introduces a bend in the corresponding region and consequently leads to the separation of this region into two short helices (His155-Thr164 and Lys162-Leu171) (Fig. 3F). From a physicochemical perspective, hMGL has an amphipathic cap helix 1 whereas cap helix1 of mtbMGL is mostly hydrophobic. A positively charged Lys is positional conserved between mtbMGL (Lys152) and hMGL (Lys165) (Fig. 3C,D). In bMGL, the short cap helix 1 is composed of small hydrophobic residues. The subsequent loop region consists of polar residues and glycines (Fig. 3E). The conformational opening and closing in hMGL is largely realized by a rolling movement of cap helix 1, whereas in bMGL the opening and closing is predominantly mediated by side chain flexibility of a single residue, Ile145, in the second stretch of the cap^22^. Based on the structural similarities of hMGL and mtbMGL it can be hypothesized that conformational changes in mtbMGL are also realized by a rolling movement of cap helix 1 similar to the one observed in hMGL. Future studies displaying different cap conformations will provide experimental insights into opening and closing mechanism of mtbMGL.

The entrance to a small cavity in proximity of the active serine accommodates the glycerol moiety in our docking studies (Fig. 2) and this cavity is markedly smaller in hMGL compared to mtbMGL. Tyr181 interacts with the glycerol moiety and is conserved in hMGL (Tyr194) (Figs 2C and 3B). The side chain of Tyr194 is within hydrogen bond distance with a free glycerol observed in the crystal structure of hMGL (PDB ID: 3HJU)^25^. Glu257 in mtbMGL corresponds to Val270 in hMGL, which cannot act as H-bond acceptor due to its hydrophobic nature. Thus, it is unlikely that the short side chain of Val270 is engaged in interactions with the MG headgroup (Fig. 3B). A hole connecting the small cavity with the outside environment was found in hMGL and bMGL and was proposed to be the glycerol exit hole^23,26^. No comparable exit hole was observed in any of the molecules of the mtbMGL structure reported here.

The mtbMGL crystal structure indicates a monomeric form of the lipase as analyzed with the PISA server^32^, which is in agreement with the size exclusion chromatography of mtbMGL in solution (Fig. 1A). Monomeric forms of MGLs have also been observed experimentally for bMGL^26^ and Yju3p^33^. PISA analysis of human MGL (PDB ID 3PE6^23^,) indicates that hMGL may form a dimer in solution consistent with literature reports using gel filtration chromatography^25^. Based on these different observations, a general substrate recruitment mechanism for MGLs that requires dimerization of the lipases seems unlikely.

Despite subtle, yet distinct differences to the structure of hMGL, mtbMGL and especially the cap of mtbMGL, resembles hMGL much better than the structurally characterized MGL from a bacterial species. It awaits further studies, whether other metabolically relevant proteins from bacterial pathogens also share higher structural similarities with their eukaryotic host organism, which could be a general strategy to better utilize host substrates.

### Differences in the binding pocket of mtbMGL compared to human MGL open the possibility for specific inhibition of MGL from the pathogen *Mycobacterium tuberculosis*

**Figure 4:**
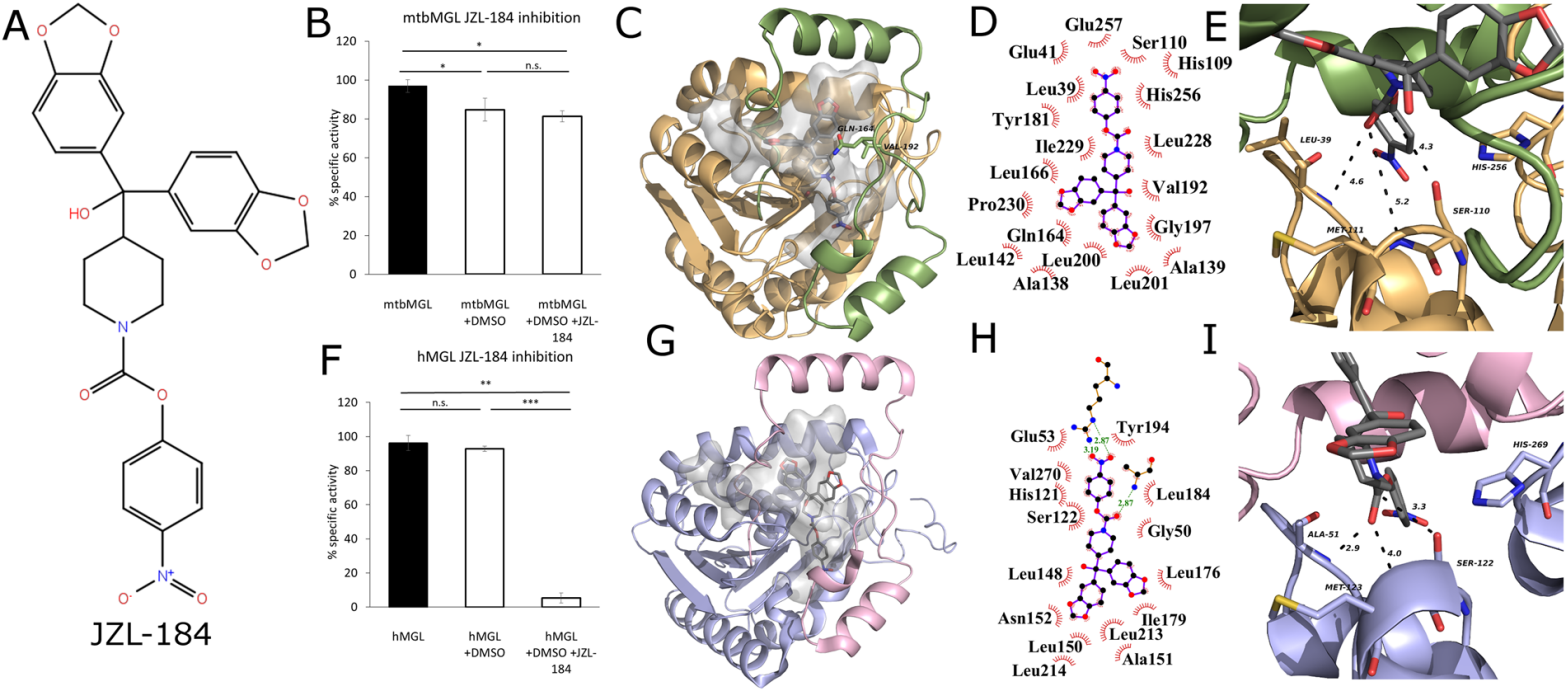
(**A**) Structure formula of JZL-184 as it was used for docking studies. The model was built using the 2D sketcher in Meastro (Maestro, Schrödinger, LLC, New York, NY, 2017). (**B**) Inhibition assays for mtbMGL and JZL-184 in DMSO and DMSO as control using 1-*rac*-oleoyl glycerol as substrate. (**C**) Cartoon representation of mtbMGL structure with JZL-184 docked. The gray blob shows the cavity inside the protein and the gray sticks the docked JZL-184. The cap is shown in green with Gln164 and Val192 restricting the cavity as sticks, core in wheat. (**D**) Ligplot+^48^ graph of JZL-184 docked to mtbMGL. The hydrophobic interactions are shown as brown circle sections. (**E**) Close up view on the active site serine and the oxyanion hole forming residues in the JZL-184 mtbMGL structure. JZL-184 is shown as gray sticks. (**F**) Inhibition assay for hMGL using JZL-184 as inhibitor and DMSO as control. (**G**) hMGL structure (PDB ID 3HJU, chain A) with JZL-184 docked. The cap is shown in pink and the core in light blue. The gray blob depicts the cavity inside the protein and the gray sticks the docked JZL-184. (**H**) Ligplot+^48^ graph of JZL-184 docked to hMGl (PDB ID 3HJU, chain A). (**I**) Close up view on the catalytic triad and the residues forming the oxyanion hole in the JZL-184 docked hMGL structure (PDB ID 3HJU, chain A) JZL-184 is shown as gray sticks.

The current therapies against tuberculosis involve a cocktail of different antibiotics and span over a period of 6–9 months. Currently, there are 10 drugs approved by the US Food and Drug Administration of which the first line anti-TB agents are isoniazid, rifampin, ethambutol, and pyrazinamide^34^. Due to the increasing prevalence of multi-drug resistant Mtb strains, there is urgent need for the development of new drugs against tuberculosis. mtbMGL emerged as a new promising drug target due to its activity during the dormant and active stage, the reported role in host lipid degradation, membrane synthesis, and the observed severely changed phenotypical appearance of the mtbMGL ortholog in *M*. *smegmatis*^17^. Independently, the high relevance of hMGL in many physiological and pathological processes has already prompted the development of different classes of inhibitors for MGLs within the last decade^28,29^. This knowledge could now be exploited in novel efforts targeting mtbMGL and thus aiding in therapeutic strategies of infected humans even in the latency phase. The compound class targeting regulatory cysteines in the hMGL cap is not attractive, since these are not conserved in the primary sequence of mtbMGL. Nevertheless, the high degree of structural similarity between hMGL and mtbMGL made us curious whether the piperidine-carbamate compound JZL-184 (Fig. 4A), a compound known to inhibit murine MGL activity with an IC50 in the low nanomolar range, would also bind to mtbMGL^35^. MGH activity assays reproduced the inhibitory effect of JZL-184 on human MGL yet exhibited no significant effect on mtbMGL (Fig. 4B).

In order to rationalize our observation, we docked JZL-184 into hMGL and mtbMGL crystal structures. The docking results for hMGL (Fig. 4G,H,I) are comparable to a previously published hMGL-JZL-184 docking study which had aimed for a covalently bound tetrahedral intermediate^25^. Our docking studies with mtbMGL revealed numerous, mostly hydrophobic interactions of JZL-184 in the substrate binding pockets (Fig. 4C–I). However, the results also indicate major differences: For hMGL the docking clearly demonstrates that there is plenty of space in the broad binding pocket of hMGL to accommodate the two arms of JZL-184, which subsequently can lead to the observed inhibition of hMGL (Fig. 4F). In case of mtbMGL, however, the cap residues Gln164 and Val192 strongly restrict the entrance of the cavity and force the inhibitor to adopt a different conformation (Fig. 4C,G). Consequently, JZL-184 cannot enter as deep into the substrate binding pocket as it does in hMGL. This restriction increases the distance between the partially positive carbon and the nucleophilic serine from 3.3 Å to 4.3 Å (Fig. 4E,I). Additionally, the carbonyl oxygen of JZL-184 is too far from the backbone nitrogens in the oxyanion hole for interaction. Together, the different orientation and penetration of JZL-184 in the substrate binding pocket of mtbMGL prevents the formation of a covalent bond between the active site serine and JZL-184 (Fig. 4E,I). Therefore, the inhibitor is free to dissociate again explaining the lack of inhibition of mtbMGL by JZL-184. In order to test the hypothesis, that JZL-184 might re-orient and eventually move towards the active site, we performed two MD simulations starting from the docking result using MAESTRO (Maestro, Schrödinger, LLC, New York, NY, 2017) (see supplementary methods). The two simulation had practically the same outcome and revealed that the distance between the active site Ser110 and the partially positive charged carbon of the inhibitor even increases and settles around 6.3 Å, which renders formation a covalent bond between the inhibitor and the serine impossible (Supplementary Fig. S2). Thus, docking studies and MD simulations for mtbMGL provide a rational explanation why JZL-184 cannot form a covalent bond to the active site of mtbMGL and fails to inhibit mtbMGL. Yet, it reaffirms the predictive power of structure-based analysis for potential inhibitors for mtbMGL. Further it enables rational design of inhibitors based on lead-compounds or modification of available inhibitors for this enzyme class.

## Methods

### Expression and purification

A synthetic codon optimized version for the gene Rv0183 from *M. tuberculosis* was ordered from Eurofins Genomics (Ebersberg, Germany). The synthetic DNA was ligated into a pProExHtb vector from Life Technologies (Carlsbad, USA) using BamHI and XhoI restriction sites. The resulting plasmid was transformed into *E. coli* BL21 (DE3) CodonPlus cells. The cells were grown in LB broth containing 100 µg/ml ampicillin. At an OD_600_ of 0.5 the Trc promotor was induced with IPTG (final concentration 1 mM) and incubated overnight at 18 °C. Cells were harvested by centrifugation and frozen at −20 °C. After thawing on ice, the cells were resuspended in buffer A containing 50 mM Tris-HCl pH 8.0 and 150 mM NaCl. After lysis via sonication, the clarified supernatant was loaded onto a gravity flow column filled with Ni-NTA resin. Unspecifically bound proteins were washed off with 10 column volumes buffer B (50 mM Tris-HCl pH 8.0, 150 mM NaCl, 40 mM imidazole). The protein of interest was eluted using 5 CV buffer C (50 mM Tris-HCl pH 8.0, 150 mM NaCl, 250 mM imidazole). Eluted protein containing mtbMGL was further purified using size exclusion chromatography (Superdex 200, GE Healthcare) in buffer A. hMGL was expressed and purified as described previously^21^.

### JZL-184 synthesis

The inhibitor JZL-184 was synthesized according to a previously published protocol^35^.

### MG hydrolase (MGH) activity assays

50 μl protein solution with concentration appropriate for activity measurement was mixed with 1 μl of JZL-184 (0.8 mg/ml) in DMSO for the inhibition assays with JZL-184^35^ or with 1 μl DMSO for the controls and incubated at room temperature for 10 min. These solutions were used directly as samples for MGH assay. The MG substrate preparation for the MGH assays was performed as described previously^21^. 660 µl of substrate solution were mixed with 5060 µl free glycerol reagent (SIGMA-ALDRICH F6428) dissolved in the same phosphate buffer as used for the substrate to get the reaction mix. 1 µl protein solution (5 ng/µl for mtbMGL and 25 ng/µl for hMGL) was mixed with 52 µl reaction mix and incubated at 37 °C for 15 min. After color development upon reaction, 6 µl stopping solution (4 M Na acetate pH 4.5) were added and the absorbance at 562 nm was quantified.

### Crystallization, data processing, and refinement

Crystallization experiments were performed with an ORYX 8 robot (Douglas Instruments, Hungerford, UK) using the sitting drop vapor-diffusion method in 96-well plates. Screening was performed using commercial screens from Molecular Dimensions (Newmarket, UK). Initial needle cluster like crystals were obtained after 4 months of incubation at 20 °C after mixing 0.5 µl protein solution (10 mg/ml) with 0.5 µl crystallization condition containing 0.1 M carboxylic acids, 0.1 M sodium HEPES/MOPS buffer pH 7.5 and 20% ethylene glycol and 10% PEG 8000 (Morpheus Screen G6, Molecular Dimensions, UK). A seeding stock was prepared from these crystals by crushing and putting them in 50 µl original mother liquor. This solution was used for micro seeding by setting up new screens and mixing 0.4 µl protein solution with 0.4 µl crystallization condition and 0.2 µl seeding stock (diluted 1:1000 with Morpheus condition G6)^36^. Using this method, a crystal diffracting to 3.5 Å was obtained in a condition containing 0.03 M NaNO_3_, 0.03 M Na_2_HPO_4_, 0.03 M (NH_4_)_2_SO_4_, 0.1 M Na HEPES/MOPS buffer pH 7.5, 12.5% MPD, 12.5% PEG 1000 and 12.5% PEG 3350. A final optimization step yielded crystals diffracting up to 1.8 Å in a condition containing 0.03 M NaNO_3_, 0.03 M Na_2_HPO_4_, 0.03 M (NH_4_)_2_SO_4_, 0.1 M Na HEPES/MOPS buffer pH 7.8, 12% MPD, 12% PEG 1000 and 12% PEG 3350. The crystals appeared after 2 weeks at 20 °C. The crystal was measured at the ESRF beamline ID23-1 (Grenoble, France)^37^. Diffraction data were indexed and integrated using Mosflm^38^. The point group was determined using pointless and the equivalent reflexes were merged using Scala^39,40^. The structure was solved by molecular replacement using the Balbes server^41^. A hybrid model of the structures with the PDB IDs 3PE6 and 1W53 was used as search template^23,42^. The initial model was completed manually in Coot^43^ and refined with Phenix^44^. Waters were placed by using the update waters option in phenix.refine^44^ and were checked manually in Coot^43^. The final structure was evaluated using MolProbity^45^. Detailed data processing and structure refinement statistics are summarized in Table 1. All structure-related pictures were generated using PyMOL (The PyMOL Molecular Graphics System, Version 1.8 Schrödinger, LLC). Structure alignment was done using the align function in PyMOL. Cavities were calculated with the PyMOL plugin CaSOXs. The atomic coordinates and structure factors have been deposited in the Protein Data Bank under the accession code 6EIC.

### Docking

#### Docking of 1-OG into the mtbMGL crystal structure

A docking grid for chain B of the mtbMGL crystal structure was generated using the Generate Grid function in MAESTRO (Maestro, Schrödinger, LLC, New York, NY, 2017) with Ser110 as center. The box sizes were 10 × 10 × 10 Å for the inner box and 40 × 40 × 40 Å for the outer box. To avoid scenarios that do not make sense for the catalytic reaction, we introduced distance restraints: The distance between the oxygen atom OG of Ser110 and the α carbon of the fatty acid was limited to a maximum of 3.5 Å the distances between the backbone nitrogens of Leu39 and Met111, which are supposed to build the oxyanion hole, and the carbonyl oxygen of JZL-184 were restrained to 3.5 Å. The model for 1-oleoyl glycerol (1-OG) was generated using the MAESTRO 2D sketcher (Maestro, Schrödinger, LLC, New York, NY, 2017). The 1-OG model was docked into the grid using the Glide docking task with precision set to “extra precision” in MAESTRO^46^. The best pose for the substrate was covalently linked to the active site serine and Force field energy minimization was done in MAESTRO using the OPLS3 force field^47^ and standard settings.

#### Docking of JZL-184 into the mtbMGL and hMGL crystal structures

Grids for mtbMGL and hMGL (PDB ID 3HJU, chain A)^25^ were generated using the Generate Grid task in MAESTRO using Ser122 as center in hMGL and Ser110 as center in mtbMGL. The box sizes for both proteins were 10 × 10 × 10 Å for the inner box and 40 × 40 × 40 Å for the outer box. The model for JZL-184 was generated via the MAESTRO 2D sketcher. JZL-184 was docked via the MAESTRO Glide docking task using extra precision and otherwise standard settings.

### Summary

We report the first structure of the MGL mtbMGL from *Mycobacterium tuberculosis* encoded by the gene *Rv0183*. It exhibits a canonical α/β-hydrolase core with a Z-shaped cap region in open conformation. Additionally, we performed computational docking studies with the natural substrate 1-OG and could identify a large network of interactions between the lipase and the ligand. We detected profound similarities between mtbMGL and hMGL, which even outrun the similarity to another bacterial MGL. Nevertheless, we could demonstrate that the existing differences between hMGL and mtbMGL are sufficient for selective inhibition. Computational docking studies of the inhibitor JZL-184 provides a rational, structure-based explanation for the observed strong inhibition of hMGL. On the other hand, results from docking and MD simulations can explain the ineffectiveness of JZL-184 on the activity of mtbMGL as a result of significant differences in the binding pockets of hMGL and mtbMGL. The structural insights open a route to rationally re-design JZL-184 and different lead compounds to synthesize specific inhibitor for mtbMGL from the pathogen *Mycobacterium tuberculosis*, while avoiding effects on the important physiological activities of human MGL.

## Electronic supplementary material

Supplementary Information
