# Supplementary material for: The crystal structure of monoacylglycerol lipase from M. tuberculosis reveals the basis for specific inhibition
Source: Sci Rep. 2018 Jun 12;8:8948. doi: 10.1038/s41598-018-27051-7 (PMC5997763; doi:10.1038/s41598-018-27051-7)
Supplement: Supplementary file 1 — Supplementary Information [file 41598_2018_27051_MOESM1_ESM.pdf]

The crystal structure of monoacylglycerol lipase from *M. tuberculosis* reveals the basis for specific inhibition

Philipp Aschauer[1], Robert Zimmermann[1,2], Rolf Breinbauer[2,3], Tea Pavkov-Keller[1], *Monika Oberer[1,2]

Supplementary Methods

<u>MD simulation of the mtbMGL JZL-184 docking result</u>

All programs for MD simulations were used via the Maestro Environment (Maestro, Schrödinger, LLC, New York, NY, 2017). Structure preparation (including energy minimization) for MD simulation was done using the Protein Preparation Wizard. We used the OPLS3 force field for minimization, the pH was set to 7.0 +- 0.2 and the simulation was carried out in an orthorhombic box filled with water. The preparation including the addition of water and Na+ ions for charge equilibration were performed with the "System Builder" panel. The MD simulation were calculated with "Desmond" employing the OPLS3 force field for 10ns with 10ps steps at 300K.

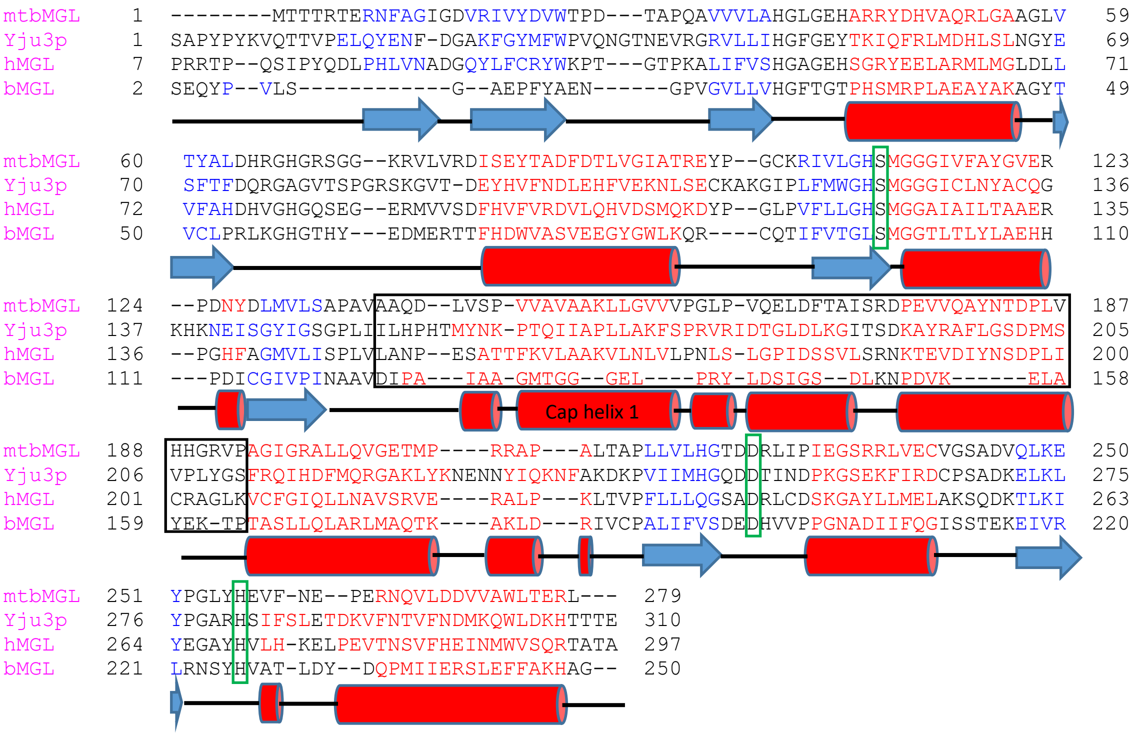

Supplementary Figure S1: Structure Based Sequence Alignment done with PRMALS3D (Pei, J., Kim, B.-H. & Grishin, N. V. PROMALS3D: a tool for multiple protein sequence and structure alignments. *Nucleic Acids Res.* **36,** 2295–300 (2008)). The sequencesof hMGL (PDB ID: 3HJU chainA), bMGL (PDB ID: 4KEA chainA) and Yju3p (PDB ID: 4ZWN chainA) were entered in form of pdb codes. The sequence of mtbMGL was copied form the UniProtKB (Uniprot entry O07427). The cap regions are indicated with a black box the catalytic triad residues with green boxes. Residues in α-helices are marked red, those in β-strands blue. The consensus secondary structure is shown below the sequences as red cylinder in case of α-helices and blue arrows in case of β-sheets.

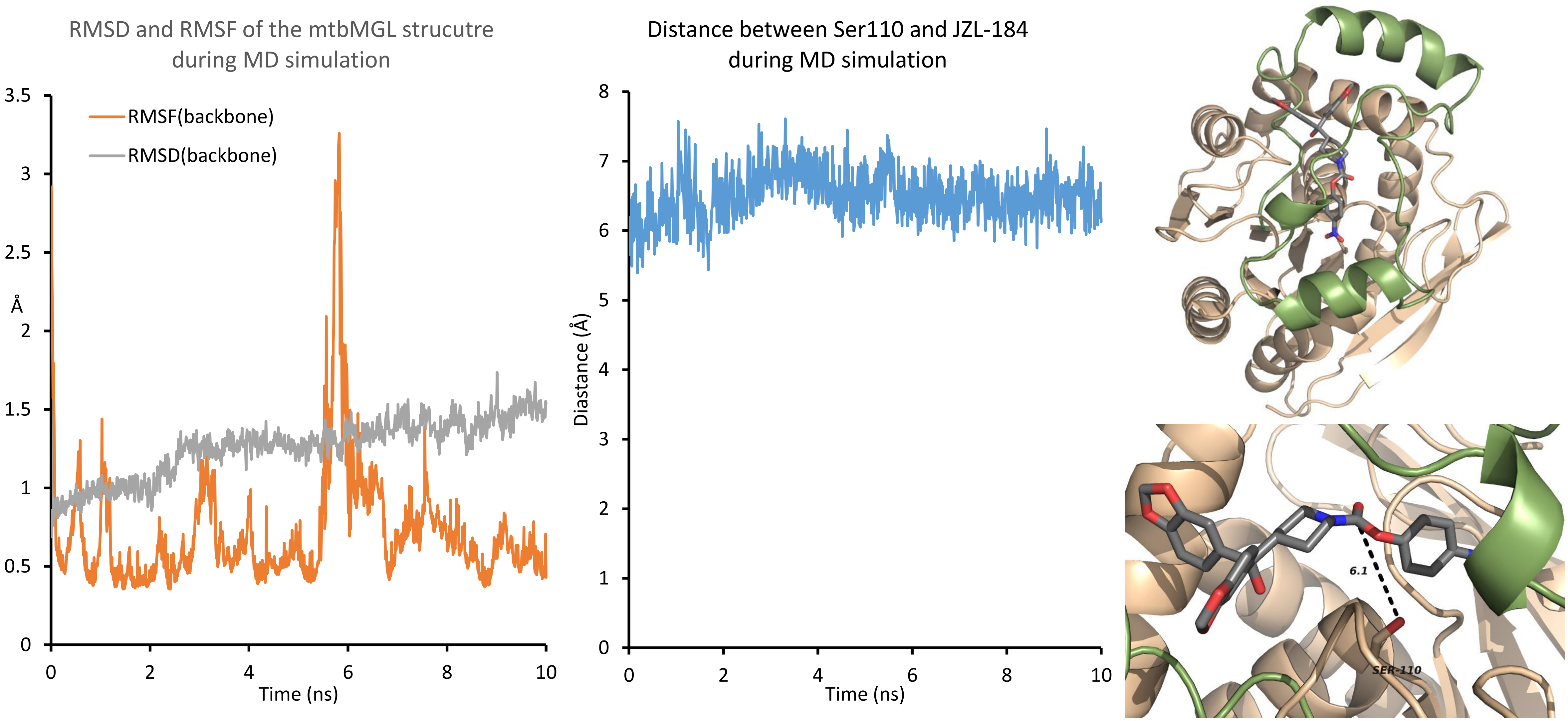

Supplementary Figure S2: MD simulation of the docking result of JZL1-84 into the mtbMGL structure. Left, root mean square deviation (RMSD) and root mean square fluctuation (RMSF) of the protein backbone compared to the energy starting structure. Middle, distance between the OG of Ser110 and the partially positively charged carbon of JZL-184 that are supposed to form the covalent bond vs. time. Right top, The overall structure of mtbMGL with JZL-184 in the last frame of the first MD simulation, JZL-184 is still inside the protein. Right bottom, a close up view on the active site Ser110 and JZL-184 in the last frame of the first MD simulation, the distance is still too long for a covalent bond.
